# Cases of Acute Articular Rheumatism, Treated in Blockley Hospital

**Published:** 1849-03

**Authors:** B. F. Wendel

**Affiliations:** Assistant Physician; Philadelphia


ART. II.—*Cases of Acute Articular Rheumatism, treated in Blockley Hospital.* By B. F. WENDEL, M.D., Assistant Physician.

CASE I.—K., aged 32 years, from Ireland, intemperate, came into my wards on the 15th December, with acute articular rheumatism, following upon a debauche, during which he had lain out for several nights. He had been subject to rheumatism during many past winters, and to wandering pains in the limbs from any sudden change in the weather, besides which his parents had been subject to the same affection. I found him with a countenance indicative of a hard drinker; a full, tense pulse; skin hot and dry, with a disagreeably hard feel; high colored and scanty urine, depositing a plentiful reddish precipitate. The affection was confined principally to the ancles, knees, wrists, and elbows, most of which were considerably swollen, especially the knees and wrists, the skin over which was tense and extremely tender to the touch. The poor fellow appeared in great distress, the slightest movement causing him to cry out with the pain.

His bowels being constipated, I ordered him four compound cathartic pills, to be followed in the morning by oil.

16th. His bowels have been freely moved without the assistance of the oil; he seems to be suffering more pain, perhaps caused by the movements of getting up to stool, and seems a little flighty. I prescribed for him nitrate of potash, ʒj, to be dissolved in a pint of water, of which he was to take a wineglassful every three hours. In the evening, his condition was about the same, and I ordered him a pill containing one grain of opium.

17th. He spent a restless night, frequently crying out. His skin is not so harsh, and his pulse is reduced slightly in force, but still continues frequent. He is ordered to continue the potash.

In the evening he was easier; he has made considerable water of a high color, and his skin is beginning to secrete.

18th. Pain in the affected joints is much abated; skin moist; tongue pretty clean; bowels moved, and altogether his condition is better. In addition to the potash, which was continued, he was ordered to be cupped on the sides of both knees, and in the neighborhood of the shoulders.

In the evening I found him resting well; pulse not so frequent, and skin moist.

19th. The cupping has had a happy effect in reducing somewhat the swelling about the joints. His urine is more copious, and presents a better color, but still depositing a plentiful precipitate. He says he is better.

From this time he continued to improve rapidly under the potash alone, until the 21st, when he told me he was purging severely, with much pain in the bowels. The pain in the joints being nearly gone, and but little swelling remaining in them, he was ordered to cease taking the potash, and use the following mixture:

℞. Ol. Ricini, ℥ij.

Tr. opii., ʒiij. M.

Of which take a teaspoonful every four hours.

In the evening he had less pain in the bowels, and they were not so frequently moved as on yesterday.

22d. The purging has nearly ceased, and the abdominal pain is entirely gone; all traces of the rheumatism have disappeared save some swelling in the knees and wrists, for the last of which he was told to go to the hydrant every morning and let the water run on the swollen joints. In three or four days the swelling had mostly disappeared, and he was able to resume his occupation.

CASE II.—S., a day laborer, aged 29 years, of temperate habits, came into the wards last evening after my visit. This morning I find him with much swelling of the knees and ancles, intense tearing pains, which he describes as being "murderous;" a very quick, frequent and hard pulse; tongue with a heavy white fur in the middle, and red at the tip and edges; eyes injected; respiration hurried; and the action of the heart tumultuous. This man was put upon low diet, and directed to take the potash in the same manner as the first one. That evening there was no perceptible change.

December 11th. But little alteration in the patient. He has a disagreeable acid smell, and the nurse informs me that he perspired greatly during the night, so much so as to moisten the sheets.

12th. He rested somewhat better last night, and feels easier this morning. His bowels are open, and there may be some reduction in the quickness of the pulse, but the frequency continues.

13th. The patient has improved. His countenance is better; his breathing but little hurried; his pulse reduced in frequency, and fuller; skin moist, but still exhaling a disagreeable odor; urine more copious, and of a more natural color. There was no farther change but for the better up to the 18th, when he was pronounced convalescent.

Case III.—K., aged 40 years, intemperate, a large, robust man, was engaged in ditching somewhere near the Schuylkill on the 12th of December, and having overheated himself, he went to the river and washed his face, and sat down on the bank for some half hour. Perspiration was thus suddenly checked, and he observed while walking home that he had taken cold. That night his cold grew worse, he was feverish, and had chilliness, with wandering pains in his limbs and body. The next day he kept his room, and was very unwell all day, the cough and pain increasing, the pain confining itself principally to the joints, which also began to swell. He came into hospital on the 14th, and I found him in the evening of that day with a very hot and dry skin; full, bounding pulse; intense headache, etc., etc. He was ordered a dose of calomel and rhubarb to open his bowels, and I deferred making a more minute examination until the morning.

15th. His bowels have been freely moved, but with no alteration of his symptoms. Seeing he had a distressing cough, I examined the chest. Percussion revealed nothing, but I could distinguish a diffused sonorous sound on putting my ear to the chest; also a slight sibillant, and I thought perhaps a slight mucous, sound. The expectoration was scanty, and what there was being a globular, whitish colored mucus, covered with a considerable frothy matter. He did not complain of any pain in the chest, but of tightness, difficulty of breathing and of expectorating. The rheumatism was confined to the knees and ancles, and muslces of the legs. The joints were considerably swollen, as also the back part of the leg. Cut cups were ordered to the sides, and after this they were to be covered with a poultice of flaxseed, and the following mixture was ordered:

℞. Decoct. senega, ℥v.

Tr. squill, ʒij.

Tr. digitalis, ʒij.

Carb. ammonia, gr.xv. M.

Of which take a tablespoonful every three hours.

For the rheumatism the nitrate of potash was ordered, and given in the same quantities, etc., as before stated.

In the evening his condition was about the same, and he was ordered not to take the cough mixture so often.

16th. Rested but little during the night. The sibillant rhonchus was not audible, only the sonorous and the mucous mingling. Does not complain so much of the tightness of the chest. Pulse continues frequent, but not so full; skin still hot and dry; bowels have been moved during the evening. Continue the same prescriptions.

17th. Pulse less frequent; skin slightly moist; urine more copious. Auscultation still reveals the sonorous and mucous sounds, but the breathing is nearly of natural frequency.

18th. Rested well last night, being only occasionally wakened by the cough. The sonorous has almost given way to the mucous râle; the pulse is reduced in frequency and force; skin pleasant; urine still more copious. He expectorates freely and easily, and the expectoration is opaque, white, and frothy. To be again cupped on the chest, and continue the prescriptions as above.

19th. Is evidently better; the tongue has cleaned handsomely, and the pulse is nearly natural, as is also the breathing. The sonorous sound is almost if not entirely gone, and the mucous rattle seems to be diminishing.

20th. Cough not very troublesome; rheumatic pains have diminished, but the swelling of the joints still continues.

21st. Expectoration not so copious; rheumatism mostly abated, and the mucous rattle is diminishing. He continues the medicines as before, but in the cough mixture, the same quantity of paregoric is substituted for the tincture of digitalis.

He continued to improve up to the 28th, when all traces of the rheumatism were gone, save perhaps a little swelling, and there remained of his disease but a slight bronchitis, which it was not thought worth while to notice.

Case IV.—L., came into my wards on December the 28th, complaining of continued pains in the elbows, knees, wrists, and ancles, from which he had been suffering for two or three days. He is a hearty German, of temperate habits, aged 25 years, who has been generally healthy, but subject to coryza from the slightest exposure.

His pains seem to be violent, but there is no swelling of the joints, and still they are tender to the touch, and quite warm. The symptoms are such as one would expect to find in an inflammatory fever, the pulse perhaps not being so full and frequent, and the urine not depositing so copious a sediment as in this form of the phlegmasiæ.

His bowels being in good condition, and wishing to try the effect of Cimicifuga in rheumatism, I ordered him to have ten grains of the powder every three hours.

It is useless to detail the daily symptoms of this patient under the use of the cimicifuga, and it will suffice to say that he continued it for a week, meanwhile taking anodynes to allay his pain, and afford him sleep, but all without avail. He was then put upon nitrate of potash, of which he took ten grains every three hours the first day, and fifteen grains every four hours the next day.

The change was soon marked in his condition; his pulse soon became natural; his skin began to act; his urine became of a more natural color; and in five days from the time he commenced taking the potash, he was almost a sound man.

Philadelphia, January 18, 1849.

